# Operative Management of Burns: Traditional Care

**DOI:** 10.3390/ebj4020024

**Published:** 2023-06-19

**Authors:** David G. Greenhalgh

**Affiliations:** 1 Burn Department, Shriners Children’s Northern California, 2425 Stockton Blvd., Sacramento, CA 95817, USA; dggreenhalgh@ucdavis.edu; Tel.: +1-916-453-2050; 2 Department of Surgery, University of California, Davis, Sacramento, CA 95817, USA

**Keywords:** burns, skin grafting, surgery, excision, skin substitute

## Abstract

Surgical treatment of burn wounds has had a tremendous impact on burn patients. The survival of patients with massive burns is now very common. Expeditious coverage of the wound has been a major contributor to improved survival, but survival is not enough. There is a need to improve the ultimate functional and cosmetic outcomes of the wound in order to facilitate a patient’s return to society. This paper reviews strategies, using fairly basic techniques, to optimize the outcomes of burn patients. While there are many new skin products available, the strategies presented here can apply to any surgeon treating burns throughout the entire world.

## 1. Introduction

Just a few decades ago, the treatment of deep burns was a passive practice that involved waiting for the burn eschar to separate followed by skin grafting onto the underlying granulation tissue. Bacteria invading and digesting the eschar were responsible for this separation while the body created a sub-eschar defensive barrier of granulation tissue. Once the eschar fell off, it was hoped that the wound bed had a low enough concentration of bacteria so as to not interfere with graft take. This chronic inflammatory process led to a profound hypermetabolic response and a high incidence of sepsis. If the wound healed, scarring was severe. Because of this drawn-out process with quite poor results, very few physicians desired to manage burns. Those who did manage burns sought improvements to this process. There were reports of excising and grafting small burns as early as the 1950s [1], but the technique did not take off. In 1970, Janzekovic, frustrated with passive wound care, decided to attempt early excision and grafting. She reported her success with this procedure in 1970 [2]. Her success led to a marked change in the strategy for dealing with deep burn wounds. Early excision and grafting have now become the norm, and burn patients have benefited greatly. Survival from massive burns is now quite routine, so the goal should focus on maximizing the functional and cosmetic outcomes. There is little benefit for the burn survivor if he or she hides at home and fears public exposure. The goal should be to have all survivors return to work or school, redevelop intimate relationships, and return to society. The goal of this paper is to provide basic strategies that optimize healing outcomes in patients with any type of burn. While there are new products available, excellent outcomes can be obtained with simple excision and autografting techniques.

## 2. Basic Skin Anatomy and Burn Depth

**Figure 1:**
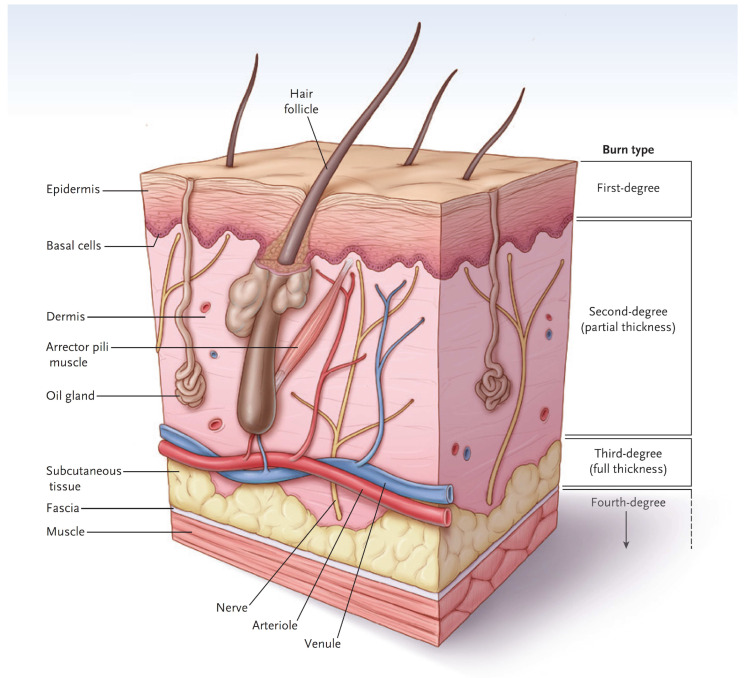
A diagram of normal skin showing the demarcations of burn types. (Reprinted with permission from Greenhalgh D.G. Management of burns. *N Engl J Med*, 2019; 380, 2349–2359. [3].)

In simple terms, the skin has two major layers, the *epidermis* and *dermis* (Figure 1) [3]. The major function of the *epidermis* is to act as a barrier to keep water inside and keep microorganisms from entering the body. The epidermis is superbly designed for this purpose. The bottom layer of cells, called the *basal cell layer*, is the only layer that has the potential for proliferation and migration over a wound. When intact, the cells of the basal cell layer differentiate and produce keratin (and other proteins) that assist with barrier function. Eventually, the outer cells undergo apoptosis and flake off. Bacterial invasion is difficult since microorganisms tend to fall off with dead cells. Since a first-degree burn does not penetrate through the epidermis, the barrier function is maintained, and the burn has a dry appearance. Melanocytes reside in the basal cell layer of the epidermis [4]. They produce melanin, package the pigment into melanosomes, and distribute the melanosomes through dendritic structures to 30–40 keratinocytes. The keratinocytes phagocytize the melanosomes and place them above the nucleus to protect them from ultraviolet light. The epidermis also contains many types of immune cells that will not be covered here.

**Figure 2:**
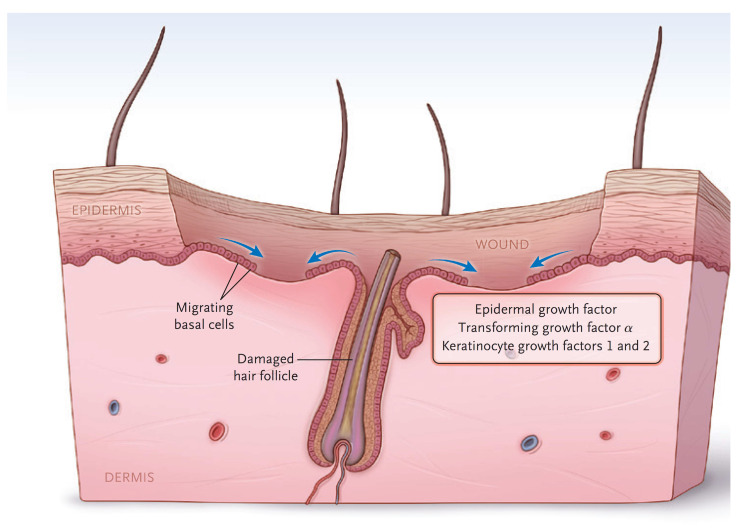
The keratinocytes migrate from skin edges and adnexa to resurface second-degree burns. (Reprinted with permission from Greenhalgh D.G. Management of burns. *N Engl J Med*, 2019; 380, 2349–2359. [3].)

The *dermis* provides the “strength” of the skin since it contains a large amount of extracellular matrix with type I collagen predominating. An epidermis, by itself, has little strength. The dermis also has a rich vascular and neural plexus that, when exposed, leads to severe pain and the release of fluids. Second-degree burns, which have lost the epidermis and weep fluids, are extremely painful, and blanch when compressed. The dermis contains invaginations from the epidermis that create the “skin adnexa”, such as hair follicles and sebaceous and oil glands. These adnexa are essential for the re-epithelialization of second-degree burns. With the loss of the epidermis, cells of the epithelial basal cell layer lose cell–cell contact inhibition and are stimulated to migrate across the viable wound surface by growth factors and proteins in the wound (Figure 2). With second-degree burns, keratinocytes migrate from the basal cells of the wound edge and up from the skin adnexa to cover the wound. Third-degree burns course through the entire dermis and into the fat. Since the protein of the skin is changed to become an eschar, the wound tends to be dry. The destruction of the dermal vascular and neural plexus leads to a wound that does not blanch and is not as exquisitely painful. In wounds lacking skin adnexa (such as with third-degree burns), migrating basal cells will travel only 1–2 cm, so unless grafted, contraction pulls the remaining edges together. Fourth-degree burns extend into structures beneath the subcutaneous fat.

## 3. Decision to Graft or Not

The most important decision is to decide whether to graft a burn or allow it to heal on its own. Clearly, this decision is based on burn depth, but it is also influenced by the size and location of the burn. In 1983, Deitch, et al. published a paper that showed that burns that re-epithelialized within 2–3 weeks had a much lower tendency to develop hypertrophic scarring than wounds that took longer to heal [5]. This finding has subsequently been confirmed by the group in Birmingham, United Kingdom [6]. There are some burn wounds that are of indeterminant depth that make it difficult to predict if they will heal within that time period. Some surgeons will wait 2–3 weeks and, if the wound has not healed, will then excise and graft the wound. While the time of healing is one factor, the decision to graft requires more thought than the timing of wound closure. Small but deep areas may be better left to contract in some areas of the body. For instance, scald burns to the chest, which are common in small children, may have small, isolated deep spots, so the decision to graft may not be appropriate. Small grafts in the middle of the chest are always visible and typically ugly. One must decide whether to wait for delayed closure and deal with a small scar or deal with an obvious graft. Clearly, one must guess which scar will be less noticeable.

Another consideration is the laxity of the skin surrounding the burn. All grafts tend to contract, and the extent of contraction increases with increased laxity of the surrounding skin. For example, a small graft on the loose skin of the inner aspect of the upper arm tends to contract more than the tighter skin of the outer arm. Skin thickness and the density of the skin adnexa of the donor site are also important. The loose, atrophic skin of the elderly often lacks hair follicles. Therefore, donor site morbidity can be significant in these patients, so allowing the burn to contract may be the better option. No decision is absolutely correct, which is why the decision to operate or not takes experience. There are newer devices such as laser Doppler imaging, indocyanine green fluorescence, and infrared thermography that may assist with the determination of burn depth that will not be covered here, but there are excellent reviews available [7,8,9,10,11]. These devices have been around for many years and they help with the decision to graft, but unfortunately, with larger burns, there may be areas that have mixed deep and superficial burns. If one just grafts the deeper areas, the resulting patch-like appearance is often cosmetically inferior to placing one large graft. On occasion, these spotty wounds that “had a chance” to heal on their own become hypertrophic right next to flatter skin grafts. Many of these decisions are difficult and require experience.

### Principles of Skin Grafting

There are some important principles that should be remembered when performing a skin graft (Table 1). There are *three phases of skin graft “take”* [12,13,14,15]. The first phase is called the phase of imbibition, where the graft survives by absorbing (imbibing) nutrients and oxygen from the wound bed. It is important to remember that the epidermal layer of the graft must survive for the entire graft to survive. If the epidermis dies, the barrier function of the graft is lost. Even though the dermis may take, with the loss of the barrier, inflammation will persist, and eventually the graft will break down. Any barrier between the wound bed and graft, such as hematoma, seroma, infection, inadequate excision, or placement of the graft on a nonviable surface (bone, tendon, necrotic tissue), will result in the graft dying. The phase of imbibition starts within hours of grafting and will persist until the graft vascularizes. The second phase is called the phase of angiogenesis. During this phase, new blood vessels must either grow into or connect to old vessels of the skin graft. This phase involves classic “angiogenesis” with the release of growth factors (angiogenic factors) from the ischemic skin, followed by the migration of nearby endothelial cells into the graft. A new blood supply may also form by a process called “inosculation”, where small vessels of the wound bed “hook up” to vessels of the graft [16,17,18]. Vascularization can occur quite quickly, especially in thin grafts. Since face grafts are often left open, one can observe them turn pink within 2–3 days. Thicker grafts will take longer. Any shearing during this time will lead to hematoma formation and, if the hematoma is not evacuated quickly enough, the graft will die. The last phase, called the phase of maturation, follows the same course for all wound healing. Collagen molecules must connect the graft to the wound bed to provide strength to resist shear. Initially, there is a rapid increase in collagen production that increases adherence. Later, there is a balance between collagen deposition and degradation associated with cross-linking between collagen molecules. If this balance between collagen production and breakdown becomes unbalanced, wound healing becomes abnormal. If there is a shift toward decreased production and increased degradation, the wound breaks down. If there is too much collagen synthesis compared to breakdown, then hypertrophic scarring results. Since the fibroblasts need nutrients to do their work, the graft remains pink to red as capillary perfusion is increased. Finally, after months to years, the redness eventually fades as the mature scar becomes relatively quiescent.

**Figure 3:**
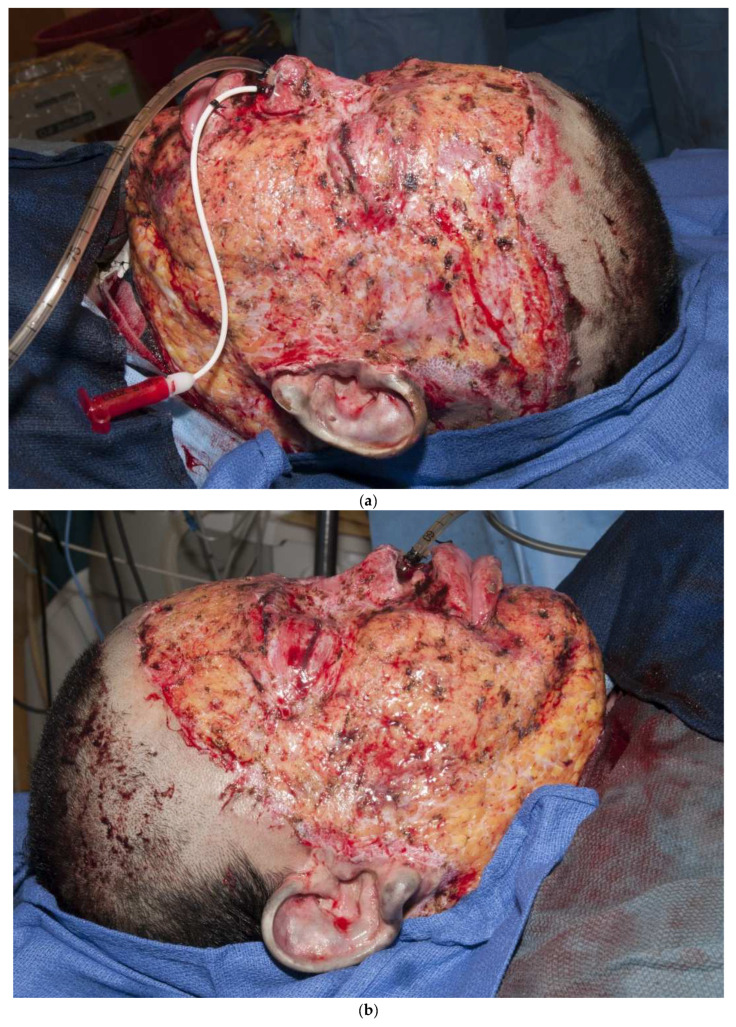

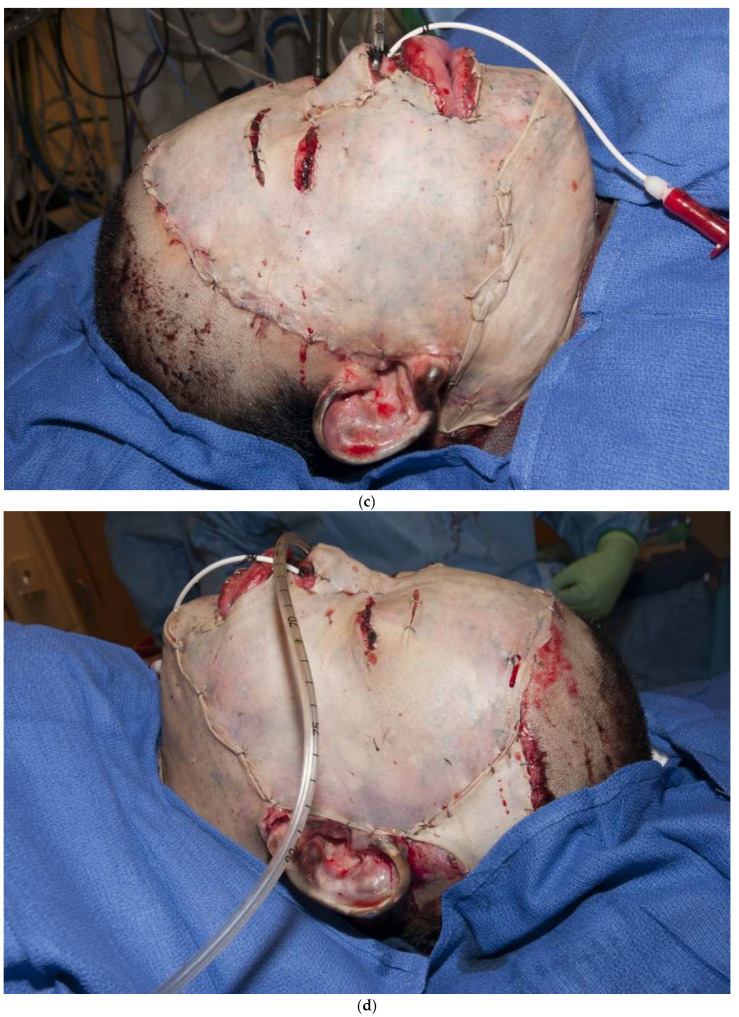
(**a**–**d**): A split-thickness skin graft can be immediately placed on fat with excellent results. This patient had a “U-shaped” sheet autograft placed around the entire face immediately after hemostasis was obtained.

Another principle is that *thicker grafts contract less than thinner grafts* [19]. It is not clear why thicker grafts shrink less, but it is likely that thicker grafts act as “splints” to counteract the “pulling” of contracting myofibroblasts. As a rule, *thicker grafts should be used in areas of more functional and cosmetic importance*. Thicker grafts should be used for hands and faces, whereas thinner grafts should be used for more proximal regions of the body. Another principle is that *“sheet grafts” usually look better and scar less than meshed grafts*. Whole sheets of skin (whether split-thickness or full-thickness) do not show the typical “mesh” pattern of meshed grafts [20]. Another important principle is that *a skin graft can immediately be placed on the freshly excised fat*. Some surgeons feel that fat will not immediately accept a graft, so a delay in coverage is required to induce inflammation or temporary allografting or xenografting is required prior to grafting. In contrast, there is no need to place a dermal substitute in order to improve the ultimate outcome. A thick sheet autograft placed immediately on fat performs well (Figure 3a–d). Finally, autografts, allografts, or xenografts all have natural proteins, called defensins or cathelicidins, that have antimicrobial activities. Skin substitutes lack these proteins and are thus less resistant to infections [21,22,23,24].

## 4. Preparation for Excision and Skin Grafting

### Pre-Operative Planning

Like all forms of surgery, pre-operative planning will lead to better results. Small grafts are a lot easier to prepare for, but one must consider where to harvest skin (see below) and whether a full or split-thickness graft is preferred. Obviously, the condition of the patient is important, but patient assessment should be the same as for any other form of surgery. There are special concerns when one prepares for the excision and grafting of a patient with large burns. It is best to have an experienced team, including anesthesiologists, circulating nurses, and scrub technicians. Open communication between team members leads to optimal outcomes. Monitoring the patient’s vital signs can be an issue. Finding sites for the placement of an oximeter when the digits are burned is difficult. They are often placed on the ears, nose, lips, or even genitalia. Electrocardiogram leads are also difficult to place, and they often need to be stapled in place.

The anesthesiologist must be prepared for potentially large amounts of blood loss and fluid shifts. Since large surface areas are exposed, blood loss can be profound. One paper described that a patient would lose around 2% of a blood volume per percent excision [25]. As an example, excision of bilateral legs would involve approximately 15–18% of total body surface area (TBSA) and could lead to loss of close to one-third of blood volume. Faces are very vascular and tend to lead to much larger amounts of blood loss per percent burn (4.5% of a blood volume per % TBSA excision). Excision of a face in an infant (~9% TBSA) could lead to the loss of nearly half blood volume. Clearly, blood needs to be typed and cross-matched prior to any excision and grafting of this extent. In addition, one must consider the need for the replacement of coagulation factors and platelets for massively burned patients. There are bleeding reduction techniques that can help (see below), but the use of tourniquets and subcutaneous epinephrine must be planned prior to surgery.

Another issue is that maintaining a patient’s temperature is more difficult in a burn patient. The skin helps to regulate temperature, and without it, hypothermia can be significant. In addition, surgery typically exposes large surface areas, both the donor and recipient sites, so regulating temperature is difficult. The room temperatures need to be uncomfortably hot for major excision and grafting procedures. There are other methods that assist with the warming of the patient such as warming devices placed on the operating room table, using warmed intravenous fluids, Bair Huggers^TM^ (3M^TM^ St. Paul, MN, USA), or intravascular warming catheters. We tend to avoid devices that blow warm air since they may increase evaporation and thus lose heat, and there is a theoretic possibility of blowing microorganisms on the wounds. Along with these issues, one must make the decision as to how much of the burn can be safely excised. It is unlikely that, despite hot rooms, a patient can tolerate more than 4–5 h of surgery. It is helpful to have a large team of surgeons when excising massively burned patients. We typically have 3–4 surgeons along with residents so that we are able to excise all extremities and anterior trunks within a few hours. The team must also ensure that they have enough allograft or other temporary wound coverage when dealing with a massive burn.

Finally, the anesthesiologist must be prepared for the special issues of burn patients. This review will only provide a few points related to anesthesia for burn patients. There are many excellent reviews on this topic [26,27,28]. The significant hypermetabolic response found in burn patients tends to cause them to rapidly metabolize medicines. In addition, since pain medication requirements persist for weeks to months, they become tolerant of narcotics and sedatives. During septic episodes, clotting factors are often consumed, so concerns for bleeding are magnified during emergency surgery. Clearly, an anesthesiologist with burn experience is essential to the best care of the patient.

## 5. Burn Excision

The concept of burn excision is simple: cut away the burn down to a viable wound bed that will “take” a graft. Like all surgery, the concept is simple, but the details are more complicated. The classic technique, called “tangential excision”, requires slicing layer after layer of the burn down to a viable wound bed. In the past, bleeding from the wound bed was used as a sign of an adequate excision. This technique would lead to excessive blood loss. In addition, bleeding does not always indicate an adequate excision. One can open larger vessels but leave nonviable surrounding tissue. One must sweep away blood using the back of the knife in order to visualize the wound bed. Fortunately, this technique is rarely practiced anymore. Most surgeons use tourniquets to minimize blood loss in the extremities. When doing so, one loses the ability to use bleeding as an indicator of the adequacy of excision. One must recognize the appearance of viable fat that has a moist and appropriate yellow color. Experience is required to recognize this appearance. To reduce bleeding in the trunk, upper extremities, and face/head, epinephrine is typically injected beneath the burn. We use 2 milligrams of epinephrine per liter of lactated Ringer’s solution. This “clysis” significantly reduces bleeding, and surprisingly, the patient tolerates the epinephrine. After excising the wound, hemostasis is required by using a cautery, ligating large vessels, and the addition of thrombin or a fibrin/thrombin agent. An autograft can then be immediately placed on the wound bed.

Another technique that is occasionally used for excising burns is to remove all burn and fat down to the fascia. The concept is that there is a better blood supply in the fascia than the fat. While there may be some truth to the blood supply, grafts take on fresh fat. Fascial excisions are severely disfiguring when compared to tangential excision and grafting on fat. Since fascial excisions lead to such functional and cosmetic problems, it is best to avoid them unless the burn depth extends to the fascia.

## 6. Full-Thickness Skin Grafts

**Figure 4:**
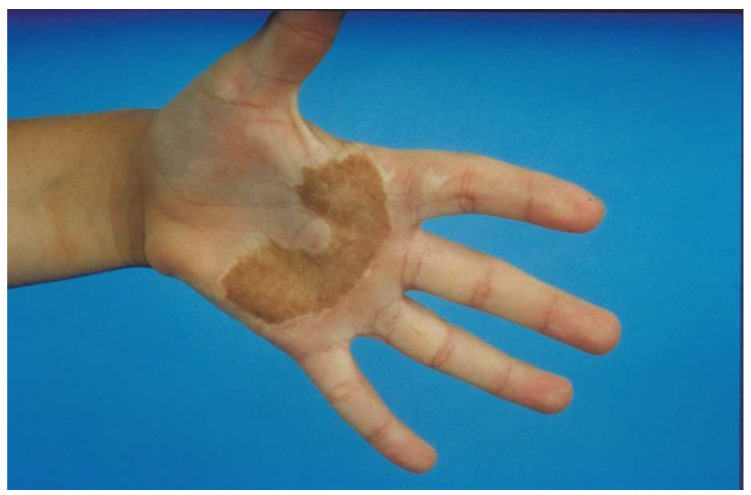
A full-thickness skin graft works well for a palm burn. The major problem with most full-thickness skin grafts to the palm is that the pigmented graft is noticeable in the pigment-free palm. An STSG from the plantar aspect of the foot has no pigmentation, but the skin is of poor quality and donor site morbidity is significant. Most patients do not mind the color change if the palm has an excellent function.

**Figure 5:**
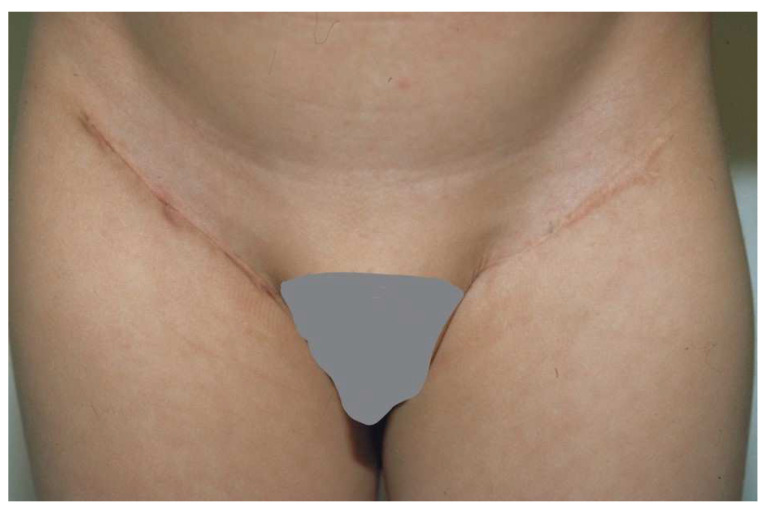
The groin is a good site for harvesting a full-thickness skin graft. The incision fits into the natural inguinal crease.

A full-thickness skin graft (FTSG) is an excellent choice for areas that need a small but thick graft. The main limitation is the size of the graft since the exposed fat of the donor site must be closed primarily or grafted with a split-thickness skin graft (STSG). FTSGs are typically used for hand (especially palm) burns (Figure 4) or smaller areas of the face (such as ectropion releases). For the hand, the inguinal crease is a good choice since a donor site large enough to cover the majority of the palm is possible. The donor site falls into the crease of the groin and is rarely seen (Figure 5). The problem with these donor sites is that they are pigmented, whereas the palm has no pigment. Some advocate the use of the sole of the foot for a color match, but the skin is of poor quality and donor morbidity is significant. These FTSGs are typically placed as outpatient surgery. The donor skin must have all fat sharply removed and if it is too thick, the dermis should be thinned. We placed the graft with sutures left long to create a “tie-over” bolster. The hand is then padded with cotton and covered with an elastic self-adherent tape (Coban^TM^, 3M^TM^, Minneapolis, MN, USA). The patient is sent home and returns for bolster removal in 1 week.

## 7. Split-Thickness Skin Grafts

### 7.1. Donor Site Selection

**Figure 6:**
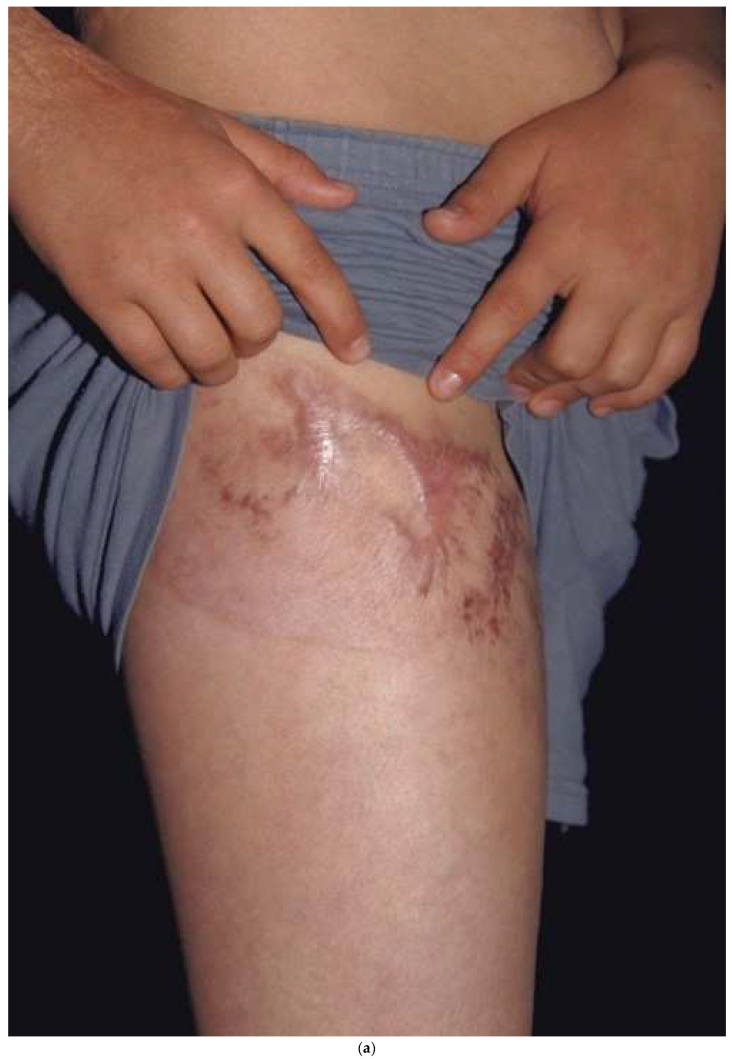

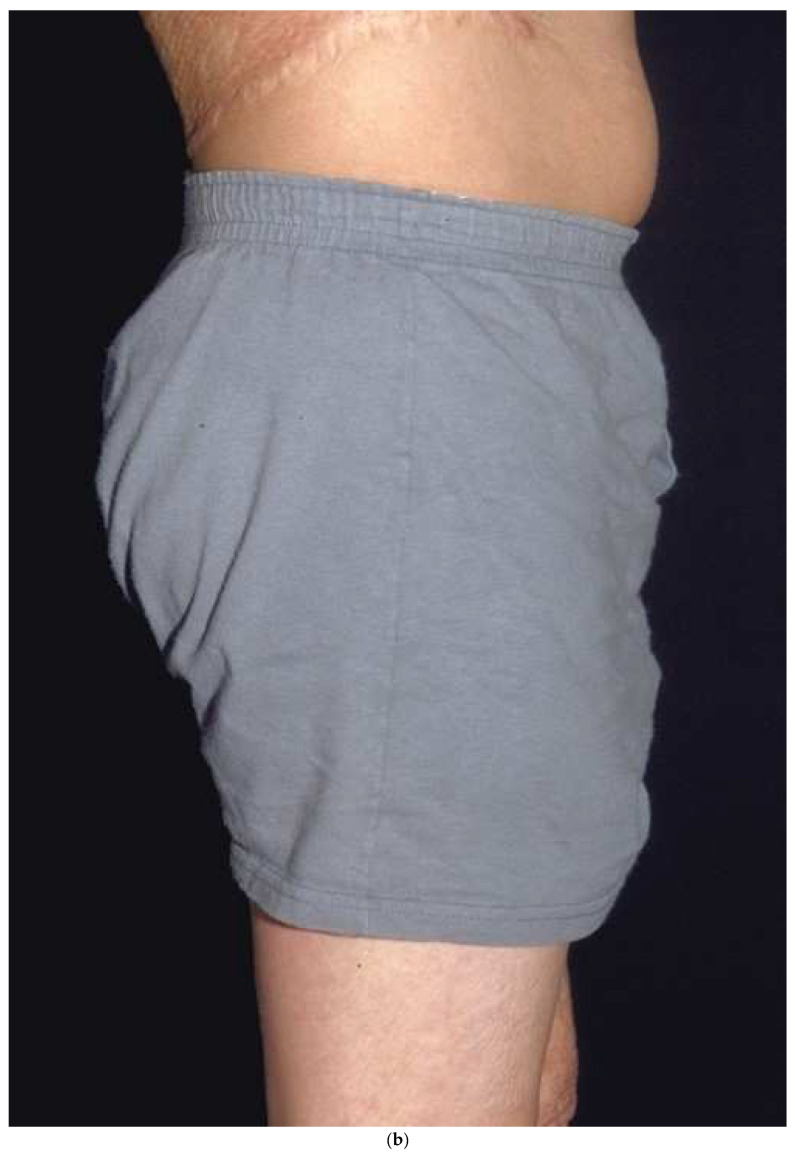
A donor site harvested in a circumferential fashion from the upper thigh (**a**) can easily be covered with shorts (**b**). If the donor had been taken down the lateral thigh it would have been very noticeable while wearing shorts.

A split-thickness skin graft (STSG) is harvested by “splitting” the donor skin through the middle of the dermis. The dermal adnexa are left behind to re-epithelialize the donor in a similar fashion as for a partial thickness burn. The goal is to obtain a thick enough STSG while allowing the donor site to re-epithelialize within 2–3 weeks. The same time-dependent relationship between donor site re-epithelialization and hypertrophic scarring exists. If the donor site has delayed healing, it is prone to hypertrophic scarring. Even with rapid healing, there tend to be some texture changes or pigmentation changes that can be noticeable. Since donor site scarring can occur, one should consider where to harvest. If, for example, one takes skin down the lateral thigh (the typical donor site) and scarring results, it will be noticeable. The patient may hesitate from wearing shorts or swimsuits. A better strategy is to harvest in the “shorts distribution” with a circumferential donor high on the thigh by starting laterally, going anteriorly, and ending medially. While the technique is a little more difficult than harvesting down the lateral thigh, the circumferential donor site can be covered with shorts (Figure 6a,b). The back has been shown to be less prone to hypertrophic scarring, probably because it has thicker skin [29]. One should try to avoid harvesting areas from the upper back or chest since these areas are exposed while wearing low-cut garments. One must remember that the density of hair follicles or other skin adnexa dictate how fast the donor site heals. The hair-bearing scalp, with a high density of hair follicles, will heal within 4–5 days. Areas of less dense skin adnexa, such as the lower legs, may take 2–3 weeks and are more prone to hypertrophic scarring. After harvest, the donor dressings should be the same as those used for superficial second-degree burns.

### 7.2. Grafting the Recipient Site

**Figure 7:**
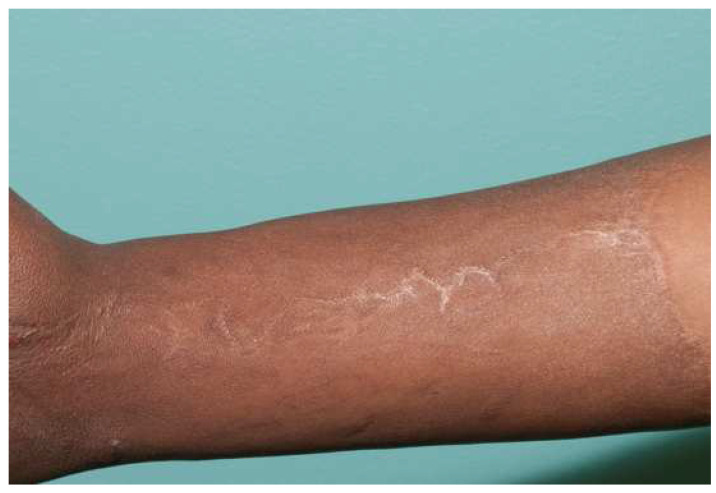
Breaking up a seam with a “Z-plasty” or dart can reduce tension. This forearm has a circumferential sheet STSG. The zigzag pattern of the seam leads to no tension in this ten-year-old graft.

There are strategies that can lead to optimal outcomes for skin grafts. For sheet grafts, all dermal elements must be removed from the recipient site, or the patient will be at risk for developing inclusion cysts, skin bridges, and pockets that fill with debris. Dr. Engrav called the result the “dreaded sponge skin” [30]. While these “sponge skin” areas can be treated by debriding the skin bridges and pockets, they can be prevented by ensuring the removal of all dermal elements. As stated earlier, thicker grafts should be placed on more functional and cosmetic areas (hands and face). If possible, the upper chest and back, especially in girls, should also be covered with sheet grafts. One must also consider any seam between a sheet graft as though it were an incision. It will also leave a line, so trying to hide the seems is important. As for an incision, long, straight seams can create tension, which leads to more hypertrophic scarring. Just as hand surgeons avoid straight incisions down fingers, seams should be broken up with darts (like pre-emptive Z-plasties) to reduce scarring [31] (Figure 7).

When there is a shortage of donor skin, or in areas of less cosmetic importance, meshing is appropriate. Meshed grafts can be placed on viable dermis with reduced risk for inclusions. On occasion, the dermis will heal, and the meshed graft will fall off. Usually, the smaller the mesh, the less contraction. The epithelial cells must migrate from the graft to fill in the interstices. On occasion, the interstices dry out or develop granulation tissue that delays healing or may lead to graft loss. The differential healing between the meshed graft and the interstices will leave a permanent meshed graft appearance. A reasonable strategy is to use spray epithelial cells (ReCell^TM^, Valencia, CA, USA), which seem to fill in the interstices more rapidly than without spray [32].

## 8. Skin Grafts of the Hand

**Figure 8:**
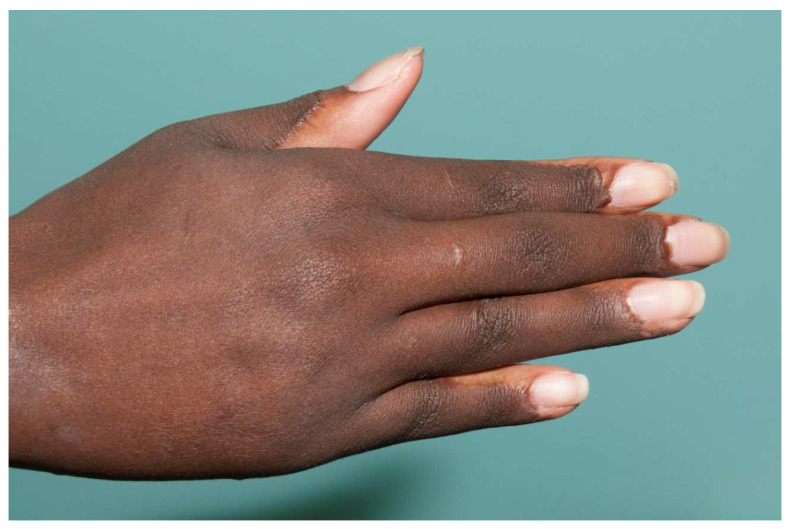
A dorsal hand burn can be covered with a single 6-inch-wide graft. The lack of any seam makes the graft look like normal skin.

**Figure 9:**
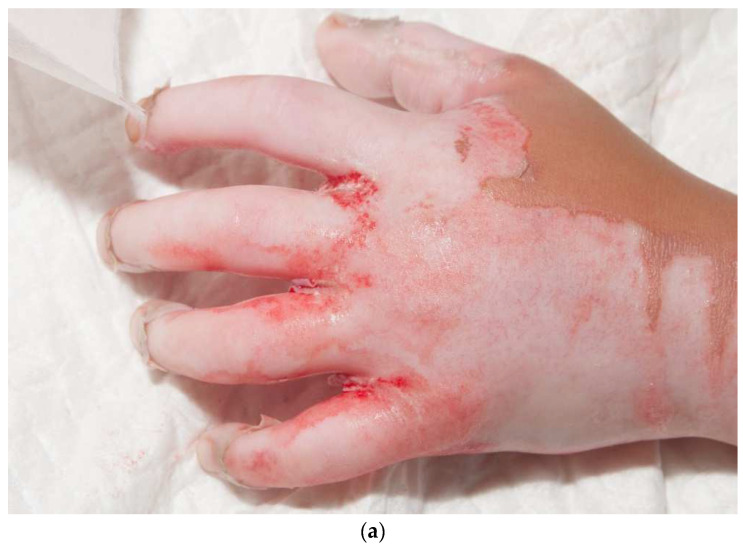

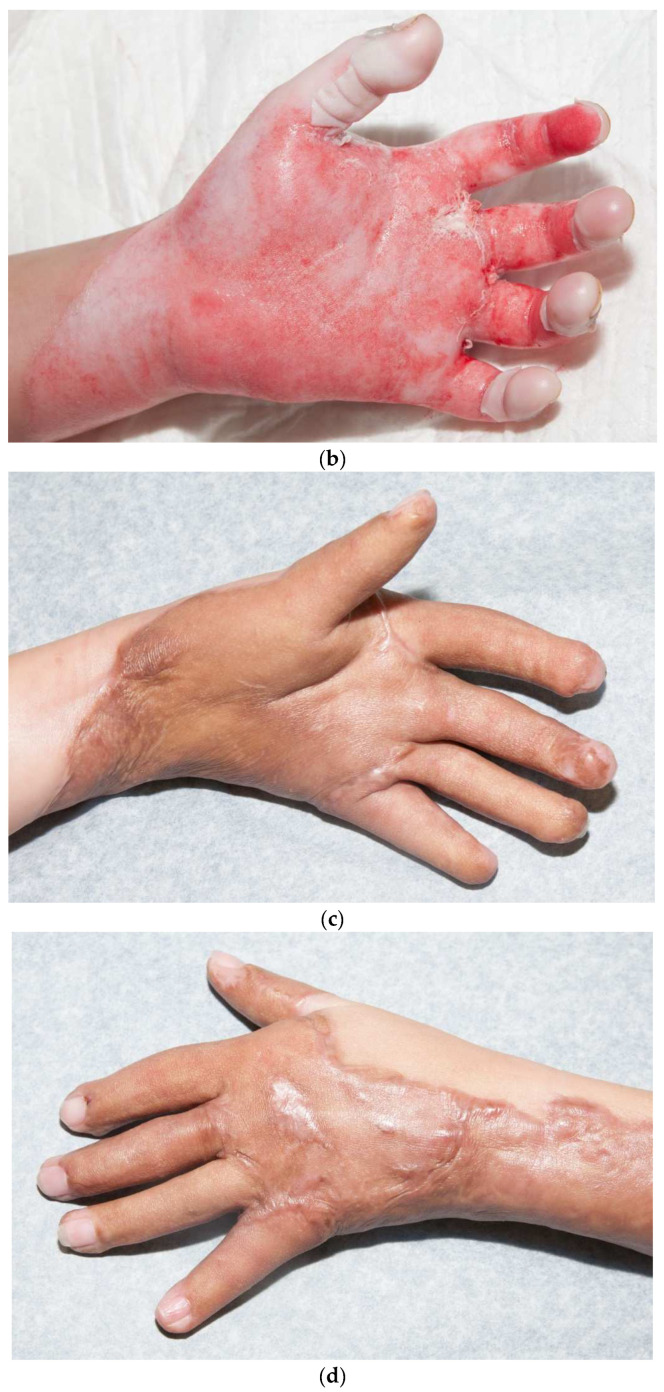
This child had a deep, circumferential burn to the left hand (**a**,**b**). A sheet STSG was used to cover the majority of the palmar hand, including all of the fingers except for the index finger (**c**). The graft was wrapped around the dorsal hand (**d**). The seams of the fingers were placed laterally on the fingers and the radial side of the thumb.

Hand skin grafts require more skill than other parts of the body since they are functionally and cosmetically important. In addition, adults have little tissue between the burned skin and deeper structures. When tendons or bones are exposed, alternatives to skin grafts may be needed. Fortunately, small children have a layer of fat in their fingers that protects these deeper structures. We use Goulian knives to excise burns from fingers, but there is an acquired skill that is required to obtain appropriate traction along with the excision. A six-inch wide dermatome can provide a wide graft that can, on many occasions, cover the entire dorsal hand without any seams (Figure 8). Covering both the dorsal and volar hand is more challenging. Typically, one piece of skin for the volar and another for the dorsal hand are required. I try to make the seams for the fingers between the second and fourth web spaces, and on the medial thumb (Figure 9a–d). Excellent grafts can be created, but they still need aggressive therapy.

## 9. Skin Grafts of the Face

**Figure 10:**
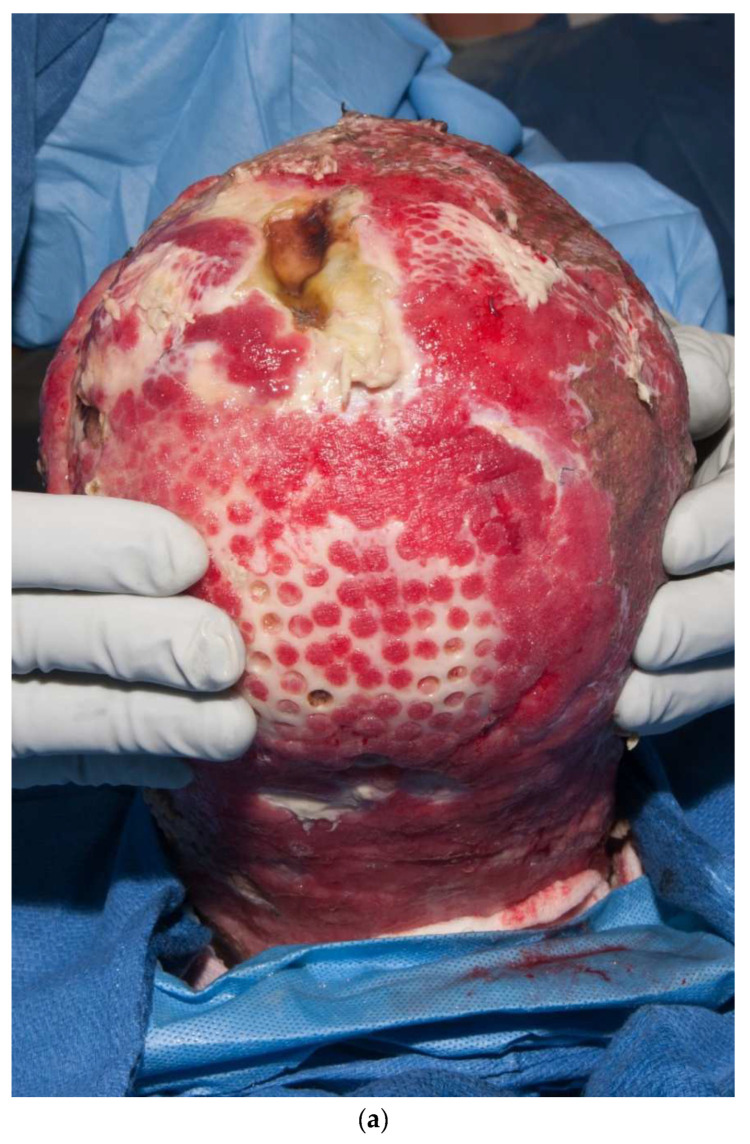

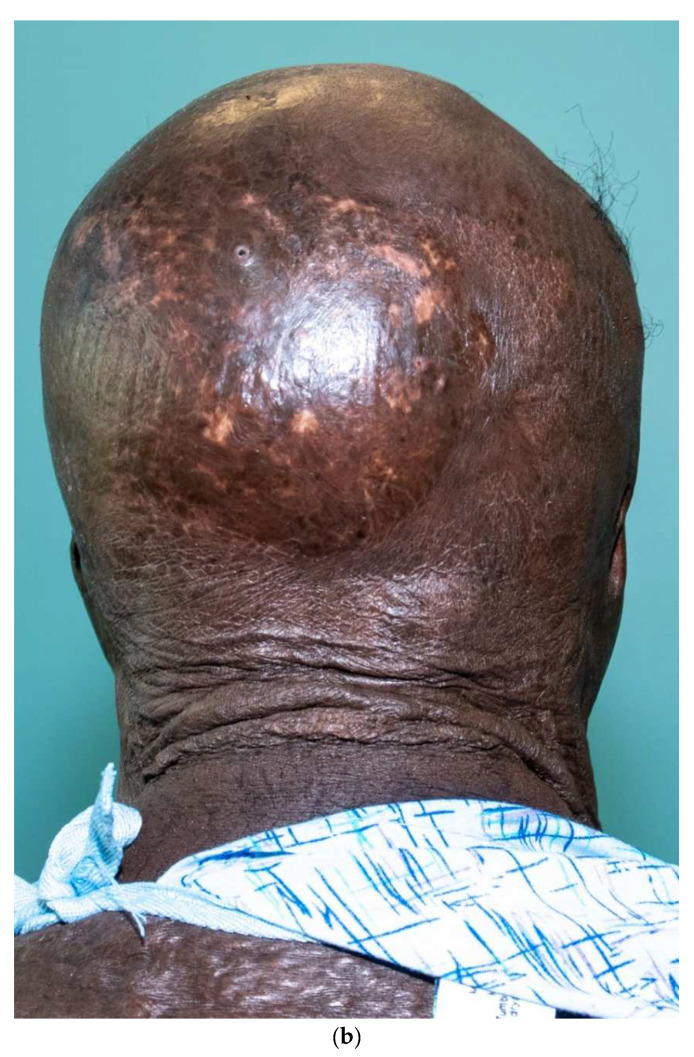
This patient had fourth-degree burns to her scalp. Since she had full-thickness burns to the upper chest, there was no available local flap to cover the defect. The outer table of the skull was burred to create multiple holes that gradually filled with granulation tissue (**a**). The scalp was subsequently grafted (**b**).

Third-degree burns on the face are also challenging. As stated earlier, the anesthesiologist must have blood available since bleeding can be significant. Injection of an epinephrine-containing solution into the subcutaneous tissue reduces blood loss to some degree, but bleeding can still be extensive. Excision of burns of the eyelids requires extra skill. Since sheet grafts should be used except in the most severe cases, complete hemostasis is required. Fibrin glues are also helpful with this process. When planning the placement of the graft, one must consider the esthetic units of the face. If possible, seams should be avoided in the middle of the forehead or cheeks. Seams should be planned for lateral to the eye or beneath the mouth. We have been harvesting circular or “U-shaped” skin in order to match the shape of the recipient site. These “U-shaped” grafts can be “wrapped” around the face to produce only one seam [33,34,35] (Figure 1a–d). If there is plenty of donor skin, grafts can be immediately placed on the fat of the recipient site with good success. If there is excessive bleeding, allograft as a temporary cover can be used. We have also placed similar grafts on dermal substitutes in patients with massive burns with good results. On occasion, the burn exposes the underlying skull, which will not “take” a graft. One option for treating these fourth-degree burns is to burr the outer table and wait for granulation tissue to form (Figure 10). If available, flaps may also be used.

## 10. Strategies for Grafting a Massively Burned Patient

There are many new strategies available to treat a massively (>40% TBSA) burned patient that will not be addressed. Only important principles will be covered here. Most burn surgeons follow the principle of early excision and grafting of patients with extensive burns. The feeling is that exposure and the nonviable tissue are major stimulants of persistent inflammation and, ultimately, the profound hypermetabolic response found in major burns [36]. In addition, it is likely that there is less bleeding with early excision and grafting than a delay of several days [37]. While the definition of early excision and closure is not clear, we typically will excise and cover the patient within 48 h of injury. With a large team, the patient can have their extremities and anterior trunk covered within a few hours. Typically, the back is excised and covered the next day, and very often the face the following day. Our typical practice is to start with autografting the hands and then cover as much of the arms as possible. For the hands, we will use sheet grafts, or if skin availability is severely limited, a small mesh is used. Wider meshed skin is then used for the arms. Some centers advocate for covering the back first, but we feel that covering more functionally important areas (hands and arms) should take precedence. One must remember that the surface area of the hands and arms is the same as the back. Once the autograft has run out, the rest of the wounds should be covered with temporary skin. In the past, we used allograft, but currently, our allograft tends to degrade within a few weeks. Dermal substitutes seem to last longer and may even slow down the hypermetabolic response. These products will be covered by others, but we currently prefer “Novosorb BTM [Biodegradable Temporizing Matrix]^TM^” (PolyNovo Biomaterials Pty Ltd., Victoria, Australia) as a temporary coverage [38]. When donor sites are available, the autograft is re-harvested to cover other areas. For massive burns (>80% TBSA), we will obtain a biopsy for cultured epithelial autograft. When the cultured epithelial autograft is ready, we will apply it over a wide (6:1) meshed autograft. We will also use ReCell^TM^ (Valencia, CA, USA) to spray epithelial cells into the interstices of a wide-meshed autograft in order to accelerate closure [32]. There are many new products and techniques available but, unfortunately, many of the dermal or skin substitutes or spray cells are expensive and may not be feasible for lower-income countries.

## 11. Conclusions

There are now excellent strategies available to cover burn wounds with the best possible functional and cosmetic outcomes. The principle of optimizing re-epithelialization so that a wound heals within 2–3 weeks should minimize scarring. When it is clear that a wound will require a longer period to heal, or if it is clearly full-thickness, grafting is usually (but not always) preferred. Thicker grafts shrink less than thin grafts, and sheet grafts eliminate the meshed pattern observed after meshed grafts. Small burns, especially on the hand, perform well with FTSGs. Larger hand burns perform very well with sheet grafts. When grafting the face, one should pay attention to placing seams that do not violate esthetic units. The use of a “U”-shaped sheet graft will minimize face seams. There are now several excellent products that allow us to cover the patient with massive burns. These basic strategies should help patients have the best outcomes and have a better chance of returning to society.

## Figures and Tables

**Table 1:** Principles of skin grafting.

• Three phases of skin graft “take”	
∘ Phase of Imbibition—starts within hours	Nutrients from the wound bed “feed” the graft
∘ Phase of Vascularization—starts at 2–5 days	Angiogenesis—migration of endothelial cells from the wound bed to the graft
	Inosculation—direct connection of wound bed capillaries to capillaries in the graft
∘ Phase of Maturation—up to 1–2 years	Collagen bonds connect the wound bed to the graft
	Eventually, the scar becomes less vascular, less raised, and improves in color
• Thicker grafts contract less than thinner grafts	
• Thicker grafts should be used on more functional/cosmetic areas (hands, face)	
• Sheet grafts look better than meshed grafts	
• Grafts can be immediately placed on freshly excised fat	
• All dermal elements need to be removed when sheet grafting	
• Straight seams create more tension and thus scar more than seams that are “broken up” by darts	
• Autografts and allografts resist infection (due to antimicrobial proteins—defensins, cathelicidins) to a greater extent than skin substitutes	
• Early excision and grafting should reduce bleeding and hypermetabolic response	

## Data Availability

This is a review so there is no data available.
